# First record of *Scolopendrellopsis* from China with the description of a new species (Myriapoda, Symphyla)

**DOI:** 10.3897/zookeys.789.27356

**Published:** 2018-10-10

**Authors:** Ya-Li Jin, Yun Bu

**Affiliations:** 1 Natural History Research Center, Shanghai Natural History Museum, Shanghai Science & Technology Museum, Shanghai, 200041, China Shanghai Science & Technology Museum, Shanghai Shanghai China

**Keywords:** antennal sensory organ, chaetotaxy, taxonomy, tergal process, Tömösváry organ

## Abstract

The genus *Scolopendrellopsis* Bagnall, 1913 is recorded from China for the first time and *Scolopendrellopsisglabrus***sp. n.** is described and illustrated. The new species is characterized by the short central rod on head, third tergite complete, four kinds of sensory organs present on antenna, and the cerci rather short and covered with a low number of straight setae.

## Introduction

There are 204 symphylan species known in the world to date (Szucsich and Scheller 2011; Domínguez Camacho and Vandenspiegel 2012; Bu and Jin 2018); however, only few publications deal with those from Asia. Hansen firstly described five species of Symphyla from Southeast Asia (Hansen 1903). After that several species were described from India (Scheller 1971), Indonesia (Scheller 1988), USSR (Scheller and Golovatch 1982), Russian Far East (Scheller and Mikhaljova 2000) and Iran (Scheller et al. 2011). Symphyla is poorly studied in China with only *Hanseniellacaldaria* from Zhejiang province and *Geophilellaorientalis* from Hebei province recorded (Zhang and Wang 1992; Bu and Jin 2018). Three genera, *Scutigerella*, *Scolopendrelloides*, and *Symphylella*, were also mentioned for China, but without determined species recorded (Zhang and Wang 1992). During our ecological survey of soil animals of Zhejiang, Jiangsu, and Hainan provinces in recent years, many symphylans were obtained. Among them, one new species of *Scolopendrellopsis* was identified and is described in the present paper.

## Materials and methods

Most specimens were collected during a project for soil animal survey of Gutian Mountain of Zhejiang Province during the years 2012 to 2013; others were collected in Jiangsu province and Hainan province recently. All were extracted by means of the Tullgren funnels from soil and humus samples and preserved in 75% ethanol. They were mounted under slides using Hoyer’s solution and dried in an oven at 60 °C. Observations were made with a phase contrast microscope (Leica DM 2500). Photographs were taken by a digital camera installed on the microscope (Leica DMC 4500). Line drawings were drawn using a drawing tube. All specimens are deposited in the collections of Shanghai Natural History Museum (**SNHM**) and Shanghai Entomological Museum (**SEM**), Shanghai, China.

## Taxonomy

### Family Scolopendrellidae Bagnall, 1913

#### Genus Scolopendrellopsis

Taxon classificationAnimaliaSymphylaScolopendrellidae

Bagnall, 1913 new record

##### Diagnosis.

Habitus slender. First pair of legs present, 3-segmented and with claws, not more than one-half length of the following pairs. Trunk with 16 or 17 tergites and most of tergites with a pair of posterior processes, without any striped band between each pair of processes on tergites, some tergites transversely divided.

##### Distribution.

The genus *Scolopendrellopsis* includes fifteen species and is subcosmopolitan, widely distributed in Palaearctic, Nearctic, Neotropical, Ethiopian, Oriental, and Australian regions (Szucsich and Scheller 2011). It is newly recorded from China in this paper.

#### Scolopendrellopsis glabrus sp. n.

Taxon classificationAnimaliaSymphylaScolopendrellidae

http://zoobank.org/95E5B444-5DEF-49CB-A699-E9730BD69528

**Figure 1.**
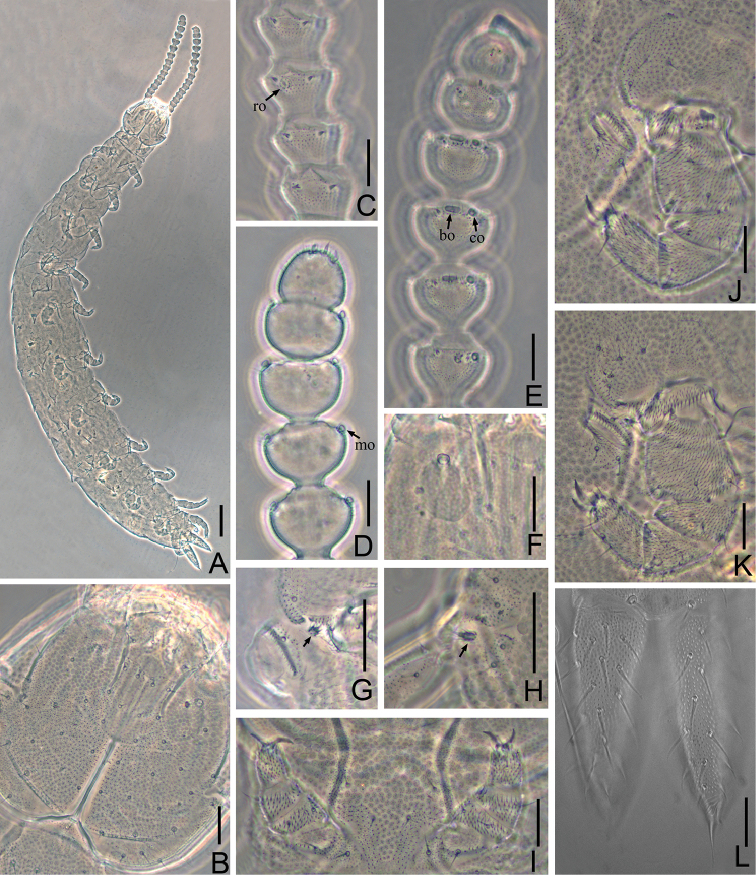
*Scolopendrellopsisglabrus* sp. n. (Holotype) **A** habitus **B** head, dorsal view **C** right antenna, 3^th^–6^th^ segments, dorsal view **D** right antenna, 12^th^–16^th^ segments, ventral view **E** right antenna, 11^th^–16^th^ segments, dosal view **F** left Tömösváry organ **G** stylus on base of 6^th^ leg (arrow indicated) **H** stylus on base of 11^th^ leg (arrow indicated) **I** first pair of legs **J** 3^rd^ leg and coxal sac **K** 9^th^ leg and coxal sacs **L** cerci, dorsal view. ro-rod-like sensory organs with surrounded setae, co-cavity-shaped organ, mo-mushroom-shaped organ, bo-bladder-shaped organ. Scale bars: 100 μm (**A**), 20 μm (**B–L**).

**Figure 2.**
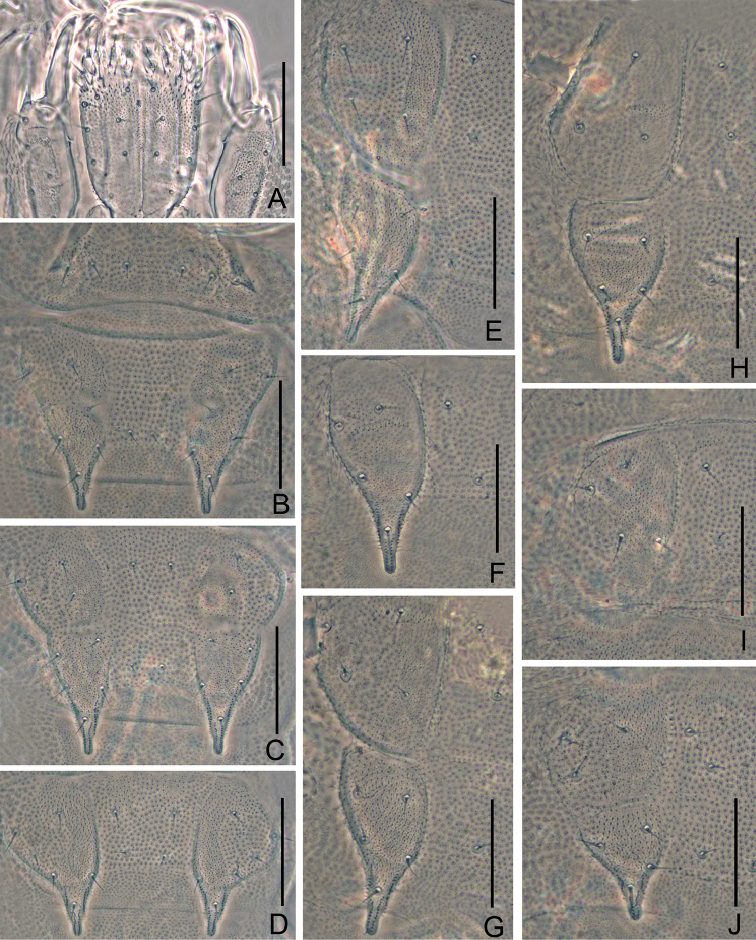
*Scolopendrellopsisglabrus* sp. n. (Holotype) **A** first and second maxilla **B** 1^st^ and 2^nd^ tergite **C** 3^rd^ tergite **D** 4^th^ tergite **E** 6^th^ tergite, left side **F** 8^th^ tergite, left side **G** 9^th^ tergite, left side **H** 12^th^ tergite, left side **I** 14^th^ tergite, left side **J** 15^th^ tergite, left side. Scale bars: 20 μm.

**Figure 3.**
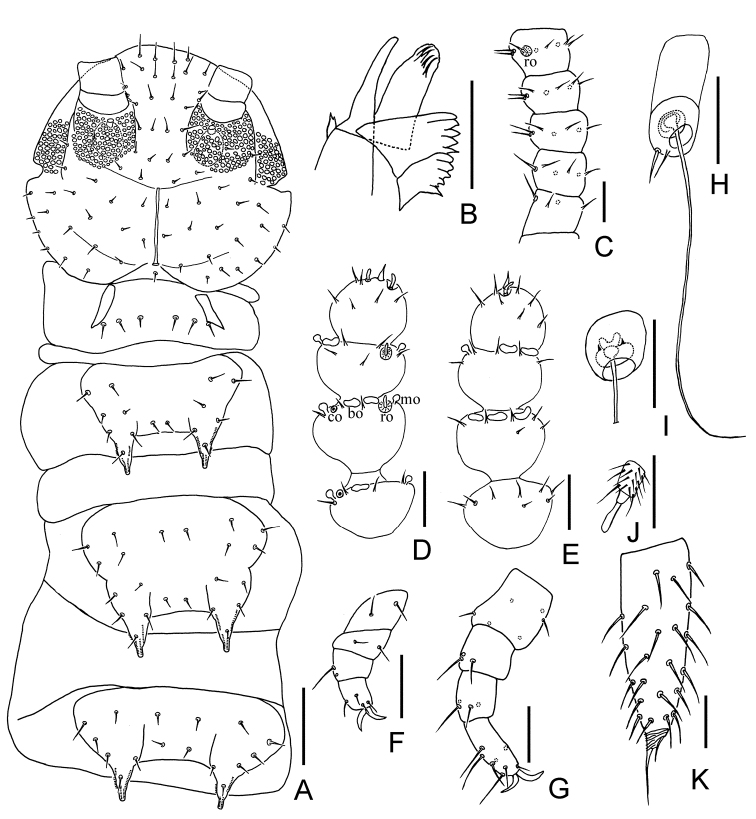
*Scolopendrellopsisglabrus* sp. n. (Holotype) **A** head and 1^st^–4^th^ tergites **B** mandible and first maxilla **C** 1^st^–5^th^ segments of right antenna **D–E** 13^th^–16^th^ of right antenna **D** dorsal view **E** ventral view **F** first leg **G** 12^th^ leg **H** left sense calicles, dorsal view **I** right sense calicles, dorsal view **J** stylus on base of 11^th^ leg **K** right cercus, dorsal view. Scale bars: 50 μm (**A**), 20 μm (**B–I, K**), 5 μm (**J**).

Figs 1 , 2 , 3 . Tables 1 , 2 , 3

##### Diagnosis.

*Scolopendrellopsisglabrus* sp. n. is characterized by the short central rod on head, 3^rd^ tergite not divided and with only weak middle indentation, rod-like sensory organs with setae surrounded on dorsal side of 3^rd^–17^th^ antennal segments, cavity-shaped organs on dorsal side of subapical 5–6 antennal segments, mushroom-shaped organs at lateral side of subapical 4–7 segments and bladder-shaped organs on subapical 3–6 antennal segments, first pair of legs longer than the tarsus of the last pair of legs, cerci short and covered with a low number of straight setae.

##### Material examined.

***Holotype***, female (slide no. ZJ-GTS-SY2012017) (SNHM), China, Zhejiang Province, Gutian Mountain, extracted from soil samples in broad-leaved forest, Alt. 1000 m, 29°15'N, 118°06'E, 11-IV-2012, coll. Y. Bu et al. ***Paratypes***, 2 female (slides nos. ZJ-GTS-SY2012010, ZJ-GTS-SY2012016) (SNHM), same date as holotype; 1 female (slide no. ZJ-GTS-SY2012051) (SEM), ibidem, 14-X-2012; 2 females (slides nos. ZJ-GTS-SY2012055, ZJ-GTS-2012060) (SNHM), ibidem, 17-XI-2012; 1 female (slide no. ZJ-GTS-SY2013015) (SNHM), ibidem, 24-IV-2013; 1 male (slide no. JS-WX-SY2017001) (SNHM), China, Jiangsu Province, Wuxi, Daji Mountain, extracted from soil samples in bamboo forest, Alt. 5 m, 31°32'N, 120°12'E, 9-X-2017, coll. Y. Bu. ***Other material*** (SNHM): 8 juveniles with 8–10 pairs of legs (slides nos. ZJ-GTS-SY2012002, ZJ-GTS-SY2012004, ZJ-GTS-SY2012006, ZJ-GTS-SY2012012–ZJ-GTS-SY2012015, ZJ-GTS-SY2012019), same data as holotype; 1 juvenile with 10 pairs of legs (slide no. ZJ-GTS-SY2012023), ibidem, 19-VI-2012, coll. Y. Bu et al; 6 juveniles with 8–11 pairs of legs (slides nos. ZJ-GTS-SY2012028–ZJ-GTS-SY2012032, ZJ-GTS-SY2012039), ibidem, 15-VII-2012, coll. Y. Bu et al; 2 juveniles with 9 and 10 pairs of legs (slides nos. ZJ-GTS-SY2012046, ZJ-GTS-SY2012051), ibidem, 14-X-2012, coll. Y. Bu et al; 2 juveniles with 10 and 9 pairs of legs respectively (slides nos. ZJ-GTS-SY2012052, ZJ-GTS-SY2012055), ibidem, 17-XI-2012, coll. Y. Bu et al; 3 juveniles with 8–10 pairs of legs (slides nos. ZJ-GTS-SY2012064–ZJ-GTS-SY2012066), ibidem, 12-XII-2012, coll. Y. Bu et al; 1 juvenile with 8 pairs of legs (slide no. ZJ-GTS-SY2013004), ibidem, 23-II-2013, coll. Y. Bu et al; 6 juveniles with 8–10 pairs of legs (slides nos. ZJ-GTS-SY2013005, ZJ-GTS-SY2013009, ZJ-GTS-SY2013011, ZJ-GTS-SY2013013–ZJ-GTS-SY2013015), ibidem, 27-III-2013, coll. Y. Bu et al; 1 juvenile with 9 pairs of legs (slide no. HN-SY-SY2017001), China, Hainan Province, Sanya, Yalong bay tropical paradise forest park, extracted from soil samples in bamboo forest, Alt. 67 m, 18°15' N, 109°37'E, 22-III-2017, coll. Y. Bu.

##### Description.

Adult body 1.57 mm long in average (1.45–1.65 mm, n = 8), holotype 1.65 mm (Figure 1A). *Head* longer than wide, length 145–175 μm, width 133–170 μm, with widest part a little behind the middle on a level with the points of articulation of mandibles. Central rod distinct and with anterior part absent, length 45–49 μm, approximately one-third of head. Dorsal side of head covered with sparse setae of different length, longest setae (12–17 μm) located at the anterior part of head, approx. 3.0 times as long as central ones (4–5 μm). Cuticle around Tömösváry organ and anterolateral part of head with rather coarse granulation. Central and posterior part of head with dense pubescence (Figs 1B, 3A).

*Tömösváry organ* oval, maximum diameter 17.0–22.5 μm, somewhat shorter than the greatest diameter of 3^rd^ antennal segment (20–23 μm), opening at front position, with diameter (4–5 μm) approx. one-fourth of 3^rd^ segment of antennae (Figs 1F, 3A).

*Mandible* with eleven teeth and divided into two parts by a gap, with five anterior and six posterior teeth respectively. First maxilla has two lobes, inner lobe with four hook-shaped teeth, palp bud-like with two distal points close to outer lobe (Figure 3B). Anterior part of second maxilla with many small protuberances and posterior part with sparse setae. Cuticle of second maxilla covered with pubescence (Figure 2A).

*Antennae* 15–19 segments (16 in holotype), length 250–350 μm (320 μm in holotype), approx. 0.2 of the length of the body. First segment cylindrical, greatest diameter a little wider than long (20–26 μm: 16–25 μm), with four setae in one whorl, the longest seta (6–11 μm) inserted at the inner side and distinctly longer than outer ones (5–8 μm). Second segment wider (20–30μm) than long (18–22 μm), with six or seven setae evenly inserted around the segment and inner setae (6–10 μm) a little longer than outer ones (5–7 μm). Chaetotaxy of 3^rd^ segment similar to preceding ones (Figure 3C). Setae on the basal segments 1–3 are slender and on proximal and distal segments rather short. Basal and median parts of the antennae with only primary whorl of setae, in subapical segments one or two minute setae present in secondary whorl (Figure 3E). Four kinds of sensory organs present on antenna: rod-like sensory organs with setae surrounded present on dorsal side of 3^rd^–17^th^ segments (Figs 1C, 3C, 3D); cavity-shaped organs present on dorsal side of subapical 5–6 segments (Figs 1E, 3D); mushroom-shaped organs present on lateral side of subapical 4–7 segments and bladder-shaped organs on subapical 3–6 segments (Figs 1D, 1E, 3D, 3E). Apical segment subspherical, width 21–22 μm, length 19–20 μm, with 10–12 short setae and wide connection to preceding segment and with two fire-shaped and three baculiform organs present on apex (Figs 1D, 3D, E). All segments covered with short pubescence. Chaetotaxy and sensory organs of antennae are given in Table 1.

**Table 1.** Numbers of setae and sensory organs of antennae (holotype).

Segments	Nos. of primary whorl setae	Nos. of secondary whorl setae	Rod-like organ with setae surrounded	Cavity-shaped organs	Mushroom-shaped organs	Bladder-shaped organs	
						Dorsal	Ventral
1^st^	4						
2^nd^	6						
3^rd^	7						
4^th^	8						
5^th^	8		1				
6^th^	8						
7^th^	8						
8^th^	8						
9^th^	9						
10^th^	9	1	1	1	1		
11^th^	9	1	1	1	1		
12^th^	9	2		1	2	1	
13^th^	9	2		1	2	1	
14^th^	7	2	1	1	2	2	4
15^th^	8	2	1	1	2	1	3
16^th^	7	2					

*Trunk*: seventeen dorsal tergites present, with 6^th^, 9^th^, 12^th^, and 15^th^ tergites transversely divided, longer than preceding ones (Figs 2E, 2G, 2H, 2J). Intertergal zones between former and later tergites present, except for 14^th^ and 15^th^, 16^th^, and 17^th^ tergites. Tergites 2^th^–13^th^ and 15^th^ each with one pair of slender chitinous processes, slightly finger-like. Basal distances between processes are approx. the same length as their length from base to tip, which is longer than its basal width. All tergites pubescent and the margins of apical part of processes ornamented with rowed coarse granules. Apical seta on processes slightly anteriorly located and anterolateral setae slightly longer than other setae. No seta between apical and inner basal setae (Figs 2B–2H).

*Tergites*: 1^st^ tergite reduced to a narrow short plate with a pair of diagonal bands and with six short setae in a row (Figs 2B, 3A). Second tergite complete, broader than long, with two slender posterior processes, 1+1 axial setae and 7+6 lateral setae (asymmetrically lack one lateral seta in holotype, 7+7 lateral setae in all paratypes), with anterolateral setae slightly longer than others, processes approx. 1.5 times as long as broad, basal distance between processes approx. the same as long as their length (Figs 2B, 3A). Third tergite entire with weak middle indentation, broader and longer than preceding one with the ratios mentioned nearly 1.6 and 0.8 respectively, 2+2 axial setae and 9+9 lateral setae (Figs 2C, 3A). Fourth tergite broader than 3^rd^ tergite, with the ratios approx. 1.2 and 0.9 respectively, 2+2 axial setae and 6+6 lateral setae (Figs 2D, 3A). The shape and chaetotaxy of 5^th^–7^th^, 8^th^–10^th^, and 11^th^–13^th^ tergites similar as 2^nd^–4^th^ tergites. 14^th^ tergite without processes and relevant area replaced by two roundish tubercles with four setae inserted on (Figure 2I). 15^th^ tergite shorter than 3^rd^, 6^th^, 9^th^, and 12^th^ tergites, with smaller processes (Figs 2C, 2E, 2G, 2H, 2J). Chaetotaxy and measurements of tergites are given in Tables 2 and 3.

*Legs*: all twelve pairs of legs with claws.1^st^ pair of legs short, 3-segmented, length 35–45 μm, not more than the length of 2^th^ pair of legs, but longer than the tarsus (30–32 μm) of last pair of legs; femur at least 1.2 times wider than long (15–22 μm: 12–15 μm), with two setae at the outer side; tibia approx. 1.4 times wider than long (14–20 μm: 10–14 μm), with dorsal seta (8–10 μm) longer than ventral one (4–6 μm); tarsus longer than wide (12–19 μm: 10–17 μm), with four setae, three dorsal (5–7 μm) and one ventral (6–8 μm); claws simple and the anterior one a little larger and broader than posterior (Figs 1I, 3F). 12^th^ pairs of legs approx. three-fourths as long as the length of the head. Trochanter longer than wide (30–40 μm: 23–31 μm), with 6 subequal setae; femur approx. as long as wide (19–25 μm: 19–25 μm), with three setae transversely, one (10–14 μm) distinctly longer than other two (6–9 μm); tibia longer than wide (19–25 μm: 15–21μm), with four dorsal setae, of which one (10–14 μm) distinctly longer than others (6–9 μm); tarsus not more than 3 times as long as wide (30–32 μm: 11–15 μm) with 8–9 setae, of which 3 are protruding and 2 depressed, longest setae (12 μm) approx. as long as the greatest width of the joint. Claws rather curved, anterior one a little longer and broader than posterior one (10 μm: 8 μm) (Figure 3G). All legs covered with dense pubescence (Figs 1I, J, K).

*Coxal sacs* present at bases of 3^rd^–9 ^th^ pairs of legs, fully developed, each with 3 setae (Figs 1J, 1K).

*Styli* present at base of 3^rd^–12^th^ pairs of legs, reduced into small knobs with tuft of setae, on 9^th^–12^th^ legs larger than on former legs, especially on 11^th^ legs (5–6 μm), distinctly longer than anterior ones (2–4 μm) (Figs 1G, 1H, 3J).

*Sense calicles* with smooth margin to pit, length nearly two times longer than outer diameter (25–35μm: 12–16μm). Sensory seta inserted in the center of cup, extremely long, length 100–120 μm, at least 8.5 times longer than other two lateral setae (11–14 μm, 7–10 μm respectively) that inserted at the edge of cup (Figs 3H, I).

*Cerci* subuliform, short, approx. half as long as head, somewhat shorter than 12^th^ pairs of legs, length at least three times as long as its greatest width (80–95 μm: 25–30 μm), sparsely covered with long and straight setae, with the longest one (12.5–17 μm) approx. half of the greatest width of the cerci, terminal area (10–13 μm) short, with length at most half of the greatest width of the cerci and circled by 6–8 layers of curved ridges. Terminal setae length 18–20 μm, distinctly longer than terminal area (Figure 1L, 3K).

##### Etymology.

The species name *glabrus*, meaning bald, to indicate the lower number of setae on cerci.

##### Distribution.

China (Zhejiang, Jiangsu, Hainan).

##### Remarks.

*Scolopendrellopsisglabrus* sp. n. is similar to *S.hirta* (Scheller, 1971) and *S.spinosa* (Sheller, 1979) in the shape of 3^rd^ tergite which is not divided, shape of processes on tergites, shape of sensory organs on antennae. It differs from the latter two species in the absence of anterior part of central rod (anterior part present but indistinct in *S.hirta*, distinct in *S.spinosa*), chaetotaxy of the 2^nd^ and 3^rd^ tergites (with four and five lateromarginal setae in *S.glabrus* respectively, five and six in the other two species), cerci with lower number of setae (more setae in *S.hirta* and *S.spinosa*), all setae on cerci long and straight (setae on inner side of cerci slightly curved in *S.hirta*, most setae on cerci short and curved in *S.spinosa*). It is also similar to the worldwide species *S.subnuda* in the shape of the first three tergites, number of lateromarginal setae of the 3^rd^ tergite, shape and number of setae of the cerci, but differs in the absence of anterior part of central rod (anterior part present in *S.subnuda*), apical seta on processes slightly anteriorly located (rather close to the apex in *S.subnuda*).

**Table 2.** Chaetotaxy of tergites (holotype).

Tergites	Axial setae	Lateral setae
1^st^	3+3	–
2^nd^	1+1^1^	7+6^8^
3^rd^	2+2^2^	9+9^9^
4^th^	2+2	6+6
5^th^	2+2	5+5
6^th^	3+3	9+9^10^
7^th^	2+2^3^	6+6
8^th^	2+2^4^	5+5
9^th^	3+3	9+9^11^
10^th^	2+2	6+6
11^th^	2+2^4^	5+5
12^th^	3+3^4^	9+9^12^
13^th^	2+2	6+6^13^
14^th^	3+3^5^	4+4^14^
15^th^	3+3^6^	7+7^15^
16^th^	1+1^7^	2+2^16^
17^th^		5+5^17^

Notes on chaetotaxy variations: ^1^ with single middle seta (in 5 specimens) or without setae (1); ^2^ asymmetrically lack one seta (3) or lack one pair of seta (1). ^3^ with 1+1 setae (1); ^4^ asymmetrically present of one additional seta (1); ^5^ with 2+2 setae (5); ^6^ with 1+1 or 2+2 setae (4); ^7^ asymmetrically present of one additional setae (1); ^8^ with 7+7 setae in all paratypes (7); ^9^ asymmetrically lack 1 or 2 setae (2); ^10^ with 7+7 setae (1); ^11^ asymmetrically present of one additional setae (1); ^12^ asymmetrically lack one or present of one additional seta (2), or with 10+10 setae (1); ^13^ with 4+4 setae (2); ^14-16^ asymmetrically present of one additional seta (2); ^17^ asymmetrically lack one or two seta (2).

**Table 3.** Measurements of tergites and processes (holotype in brackets) (in μm).

No. of tergites	Length	Width	Length of processes	Width of processes	Basal distance between processes
1^st^	30–40 (40)	75–100 (80)	–	–	–
2^nd^	45–55 (55)	78–100 (100)	25–45 (31)	15–35 (20)	25–35 (30)
3^rd^	50–85 (82)	95–130 (112)	30–48 (40)	23–30 (25)	28–35 (33)
4^th^	45–61 (61)	100–125 (122)	30–50 (50)	30–40 (40)	35–45 (45)
5^th^	41–59 (59)	103–125 (107)	50–55 (50)	25–30 (30)	40–50 (40)
6^th^	55–123 (116)	130–150 (140)	40–58 (55)	25–35 (35)	40–55 (55)
7^th^	60–71 (71)	140–160 (160)	50–65 (50)	40–50 (50)	40–66 (66)
8^th^	55–75 (75)	110–135 (110)	50–65 (55)	25–35 (33)	58–68 (68)
9^th^	100–110 (110)	150–170 (160)	60–70 (60)	25–40 (30)	70–80 (80)
10^th^	67–71 (71)	150–187 (166)	40–70 (70)	30–50 (50)	45–74 (74)
11^th^	60–75 (68)	130–138 (138)	30–65 (50)	33–40 (33)	55–70 (60)
12^th^	75–120 (120)	150–165 (160)	54–60 (55)	25–40 (33)	55–65 (64)
13^th^	45–69 (69)	110–180 (156)	30–50 (50)	25–50 (50)	50–60 (60)
14^th^	55–80 (65)	95-140 (140)	–	–	–
15^th^	70–98 (85)	110–150 (140)	38–45 (40)	25–30 (30)	35–50 (42)
16^th^	35–43 (38)	95–130 (115)	–	–	–
17^th^	58–65 (65)	75–125 (90)	–	–	–

## Supplementary Material

XML Treatment for Scolopendrellopsis

XML Treatment for Scolopendrellopsis glabrus
